# Empirical research on Kano’s model and customer satisfaction

**DOI:** 10.1371/journal.pone.0183888

**Published:** 2017-09-05

**Authors:** Feng-Han Lin, Sang-Bing Tsai, Yu-Cheng Lee, Cheng-Fu Hsiao, Jie Zhou, Jiangtao Wang, Zhiwen Shang

**Affiliations:** 1 Ph.D. Program of Technology Management, Chung Hua University, Hsin Chu City, Taiwan; 2 Zhongshan Institute, University of Electronic Science and Technology of China, Guangdong, China; 3 Economics and Management College, Civil Aviation University of China, Tianjin, China; 4 China Academy of Corporate Governance, Nankai University, Tianjin, China; 5 Department of Technology Management, Chung Hua University, Hsin Chu City, Taiwan; 6 Department of Hospitality Management, Hsing Wu University, New Taipei City, Taiwan; 7 College of Tourism and Service Management, Nankai University, Tianjin, China; 8 Business School, Nankai University, Tianjin, China; Southwest University, CHINA

## Abstract

Products are now developed based on what customers desire, and thus attractive quality creation has become crucial. In studies on customer satisfaction, methods for analyzing quality attributes and enhancing customer satisfaction have been proposed to facilitate product development. Although substantial studies have performed to assess the impact of the attributes on customer satisfaction, little research has been conducted that quantitatively calculate the odds of customer satisfaction for the Kano classification, fitting a nonlinear relationship between attribute-level performance and customer satisfaction. In the present study, the odds of customer satisfaction were determined to identify the classification of quality attributes, and took customer psychology into account to suggest how decision-makers should prioritize the allocation of resources. A novel method for quantitatively assessing quality attributes was proposed to determine classification criteria and fit the nonlinear relationship between quality attributes and customer satisfaction. Subsequently, a case study was conducted on bicycle user satisfaction to verify the novel method. The concept of customer satisfaction odds was integrated with the value function from prospect theory to understand quality attributes. The results of this study can serve as a reference for product designers to create attractive quality attributes in their products and thus enhance customer satisfaction.

## Introduction

Product development was previously producer-oriented, but has now switched to being led by the customer. From customers’ perspective, the Kano’s model has been used to understand customer needs by identifying and classifying the quality attributers [1]. Product quality is typically determined by customers, with their satisfaction an indicator for the direction in which a product should be developed. This indicator is considered valuable by governments, and national customer satisfaction indexes have thus been established in many countries. In a fiercely competitive market, some products’ life cycles became shorter, such as consumer electronics, so understanding customers’ satisfaction levels with previous products is crucial for designing desirable products in a timely manner [2]. Within product development, attractive quality creation has become paramount, and quality engineering and management has transitioned from production-oriented to quality control-oriented, thereby satisfying customer needs [3].

Many studies on customer satisfaction have been published in recent decades. Martilla and James [4] investigated automotive services and used importance-performance analysis (IPA) to develop corporate strategies. They integrated the analysis of two dimensions (importance and performance) to evaluate quality attributes that were crucial to customers but did not result in the expected performance and thus needed to be improved. Kano et al. [1] investigated TV and lamp products, and observed that customers’ product awareness was not simply one-dimensional. Accordingly, they developed a two-dimensional quality model. They considered that quality attributes and customer satisfaction had an asymmetric and nonlinear relationship, and that for a product, its must-be and attractive quality attributes must considered in addition to its one-dimensional quality attributes. Numerous researchers have further studied Kano’s model, giving explanations about customer satisfaction [5]. Parasuraman, Zeithaml, and Berry [6] investigated the quality of services provided by banks, securities businesses, credit card companies, and product maintenance companies, and developed the Parasuraman, Ziethaml, and Berry (PZB) gap theory, which proposed five types of quality gap. A quality gap is the gap between customers’ expectations for the service and their feelings after using it; that is, between expected and perceived performance. Llosa [7] proposed the Tetraclasse model and investigated the effect of quality attributes on satisfaction to classify products by their quality. IPA and PZB gap theory analyzed quality attribute performance and customers’ quality gap, and Kano’s model and the Tetraclasse model explored the influence of quality gap on customer satisfaction. Although various methods have been adopted, they all were intended to achieve an identical purpose through the crucial quality attributes; to enhance customer satisfaction.

The two-dimensional quality model developed by Kano has been used during product development and design [8–10], and using Kano’s model to examine customers’ preference for product functions and classify quality attributes is conducive to decision analysis in a product development project. Kano’s model has previously been integrated with other tools to clarify the requirements of customers; these tools include PZB's gap theory [11], IPA [12–13], quality function deployment [14,15], failure mode and effect analysis [16], and the theory of inventive problem solving [17]. The conventional Kano’s model can only present the results of classifying quality attributes. Brandt [18] proposed penalty reward contrast analysis (PRCA) analysis, using dummy variable regression to classify quality attributes. Subsequently, dummy variables have been adopted by numerous studies to analyzed the asymmetric and nonlinear relationship between the performance of quality attributes and overall customer satisfaction [19–22]. Lin [23] reported that dummy variable regression presented skewed sample distributions, and suggested that, based on the line/curve shape within Kano’s model, moderated regression could be used to classify quality attributes. Lin [23] also indicated that, according to expert comparison, moderated regression was superior to dummy variable regression. Chen [24] concluded that dummy variable regression could not accurately classify quality attributes, and that moderated regression incorrectly classified quality attributes because of the cofounding effect between attribute-level performance and customer satisfaction. Thus, Chen [24] used ridge regression to handle the interaction between attribute-level performance and satisfaction. Table 1 provides a summary of various approaches for assessing the asymmetric and nonlinear relationships between attributes and customer satisfaction. Quality attributes often change, and Borgianni and Totini [25] asserted that current classification of quality attributes within a long-term product design project was insufficient. Anderson and Mittal [19] revealed the nonlinear relationship between attributes’ importance and customer satisfaction. In many studies, linear regression was used to fit a nonlinear relationship, thereby incorrectly classifying quality attributes and incorrectly understanding changes in quality attributes. Logistic regression is a nonlinear model, and is thus more suitable for fitting a nonlinear relationship than linear regression. Using quantified odds to assess quality attributes facilitates effectively understanding customer satisfaction.

**Table 1:** Summary of approaches.

Author	Approach	Assessment	
		The asymmetric relationship	The nonlinear relationship
Brandt [26]	PRCA Regression analysis with dummy variables	Asymmetric effects on Customer satisfaction	None
Ting and Chen [20]	Regressing analysis applying natural logarithms	The attributes performances affect the overall satisfaction asymmetrically	Point to the nonlinear relationship between attributes and customer satisfaction
Matzler et al. [27]	Regressing analysis with dummy variables	The impact of the different attributes on overall satisfaction	None
Lin et al. [23]	Moderated regression with dummy variables	Moderated effect of attribute-level on customer satisfaction	Predict relation curves between attributes and customer satisfaction
Finn [28]	Polynomial regression	Assess the shape of satisfaction response functions for classify attributes	Test nonlinear effects of quality attributes on customer satisfaction
Chen [24]	Ridge regression	Confirm the asymmetric customer satisfaction effects, and classify quality attributes, including mixed-class distribution	Explore the nonlinear customer satisfaction effects

Witell, Löfgren, and Dahlgaard [29] indicated that a large number of studies have applied Kano’s model without exploring the implications of attractive quality. Thus, few studies have investigated attractive quality creation and the life cycles of quality attributes [29]. The development of Kano’s model has facilitated modifying questionnaire design and classification methods and analyzing the influence of quality attributes on customer satisfaction. However, if the asymmetric and nonlinear relationship between quality attributes and customer satisfaction proposed by Kano is not adequately explored, the risk of customer dissatisfaction due to inadequate quality attributes’ performance is not decreased and the odds on customer satisfaction is not enhanced. Wang & Wu [30] incorporate combining conjoint analysis and Kano model to optimize product varieties. However, conjoint analysis has been employed to capturing customer preference, but the use of the Kano classification as a research field has not yet been much explored. Besides, Jung, Sydnor, Lee, and Almanza [31] argued that conjoint analysis excludes a priori the possibility of using a lexicographic decision-making rule and employed logistic regression to explore the effects of attributes on customers’ choices. The skew responses of dummy variable regression affect the result [24]. Unlike linear regression, the assumption of normality are unnecessary for logistic regression. The logistic regression is a direct probability model, and the odds of logistic regression are estimated conditional on the independent variables [31–32]. Therefore, logistic regression is recommended for analyzing risk and odds. Few studies have explored customer psychology and the implications of attractive quality attributes. Therefore, this study was aimed to explore the odds on customer satisfaction due to high quality performance and the risk of customer dissatisfaction due to low quality performance, therefore enhancing quantitative assessment and classification criteria through fitting nonlinear relationships. Based on customer psychology, this study also investigated how decision-makers should prioritize the allocation of resources. By quantitatively assessing quality elements that enhanced customer satisfaction, and by objectively classifying quality attributes, this study clarified the asymmetric and nonlinear relationship between quality attributes and customer satisfaction, and determined how resources could be allotted to improve quality. Attractive quality is based on customers’ cognition; understanding customers facilitates the identification of efficient quality attributes that would require few resources to improve.

## Literature review and conceptual background

### Kano’s model and customer satisfaction

The relationship between quality attributes and customer satisfaction is asymmetric and nonlinear. Herzberg, Mausner, and Snyderman [33] proposed the motivation-hygiene theory describing job satisfaction in the workplace (also called the two-factors theory). Hygiene factors refer to factors that eliminate job dissatisfaction, whereas motivation factors refer to factors that give rise to job satisfaction. Swan and Combs [34] proposed a two-factors theory, elucidating that the instrumental and expressive dimensions influenced customer satisfaction and dissatisfaction, respectively; therefore, that satisfaction and dissatisfaction were independent of each other. Kano et al. [1] classified five types of quality attribute that lead to customer satisfaction and dissatisfaction when the attributes are sufficient or insufficient, respectively; they are must-be, one-dimensional, attractive, indifferent, and reverse quality attributes.

1. Must-be quality attributes: sufficient quality attributes *do not* lead to customer satisfaction, but insufficient quality attributes lead to customer dissatisfaction.
2. One-dimensional quality attributes: sufficient quality attributes lead to customer satisfaction, and insufficient quality attributes lead to customer dissatisfaction.
3. Attractive quality attributes: sufficient quality attributes lead to customer satisfaction, but insufficient quality attributes *do not* lead to customer dissatisfaction.
4. Indifferent quality attributes: sufficient quality attributes *do not* lead to customer satisfaction, and insufficient quality attributes *do not* lead to customer dissatisfaction.
5. Reverse quality attributes: sufficient quality attributes lead to customer dissatisfaction, and insufficient quality attributes lead to customer satisfaction.

The original Kano questionnaire consists of functional and dysfunctional questions for each attribute. The highest response frequency determines the kano category through a special evaluation table [1].

Based on Kano’s two-dimensional quality model, Brandt [26] proposed a three-factors theory to classify quality attributes into minimum-requirement, value-enhancing, and hybrid attributes, and numerous studies have verified the theory [19, 27, 35, 36]. Matzler et al. [27] classified quality attributes into three types: basic, excitement, and performance factors. As a must-be quality, basic factors unidirectionally influenced customer dissatisfaction; as an attractive quality, excitement factors unidirectionally influenced customer satisfaction; and as a one-dimensional quality, performance factors were common factors whose absence led to dissatisfaction and whose presence led to satisfaction. The relationship between quality attributes and customer satisfaction was thus not represented as a symmetric and linear relationship, but an asymmetric and nonlinear relationship.

### The relationship between quality attributes and customer satisfaction

While Kano’s model analyzed satisfaction qualitatively, quantitative definitions require further investigation [2]. Recently, research has been performed on the asymmetric and nonlinear relationship between quality attributes and customer satisfaction [29, 37] using Kano’s two-dimensional quality model. Numerous studies have explained how various types of quality attribute influenced customer satisfaction distinctly, but have not indicated differences among various quality elements within one quality attribute; consequently, insufficient information is provided to enable decision-makers to identify crucial quality attributes [29]. Shahin et al. [2] precisely classified quality attributes and considered that the influence of quality attributes on customer satisfaction could be understood according to the importance level of the attributes. Wang and Ji [38] employed a S-CR (customer satisfaction and the fulfillment of customer requirements) relationship function in the analysis of Kano’s model. Finn [28] used prospect theory to determine the quality attribute line/curve shape and customer satisfaction. Some studies have applied a back-propagation neural network to analyze the causal relationship between quality attributes and customer satisfaction [39]. Fuzzy theory has also been used to classify quality attributes [40]. Compared with Kano’s model, some studies have performed a more detailed quantitative analysis and determined the importance of quality elements in a single quality attribute, verifying an asymmetric relationship between quality attributes and customer satisfaction. However, the nature of this nonlinear relationship remains to be investigated [41–42].

In recent years, the number of studies using Kano’s model has substantially increased, but its quantitative evaluation, classification criteria, and decision support still need to be improved [2, 29, 43–45]. Some researchers who adopted Kano’s model could not identify attractive qualities because of an inadequate questionnaire design, inappropriate quality attribute definition, or improper quality attribute life cycles [46–47]. Improvements to Kano’s model have been proposed, for example modifying questionnaires, improving classification methods, or integrating Kano’s model with other tools [12, 44, 48–50]. A quantitative evaluation explains the importance of each quality attribute to customer satisfaction. Such evaluations have been previously performed using classification rules to explain the asymmetric relationship between quality attributes and customer satisfaction [20, 51–53]. One study on product development integrated Kano’s model with producer’s capacity [44]. Other studies have considered customer psychology, and suggested that before attractive quality attributes are improved, must-be quality attributes or one-dimensional quality attributes should be improved [54]. Questions such as whether overall customer satisfaction improves if quality attribute performances improves, and whether overall customer satisfaction deteriorates if quality attribute performances deteriorates, merit further investigation.

### Psychological perspective

From a cognitive psychology perspective, Kahneman and Tversky [55] adopted the concept of a value function and established the psychological codes of gain and loss. They used the concept of transaction utility to construct a purchase model, and proposed prospect theory. According to prospect theory, people’s preference is generally nonlinear. The three basic principles of the theory are as follows [56]:

1. Most people tend to avoid risk when facing “gain.”
2. Most people tend to prefer risk when facing “loss.”
3. People are more sensitive to loss than to gain.

According to prospect theory, overall customer satisfaction is sensitive to quality attribute performance, and decreases if the performance decreases. The impact of negative attribute performance on overall customer satisfaction was determined to be greater than the impact of the positive attribute performance [51, 54]. Mittal, Ross, and Baldasare [51] adopted prospect theory to explore the influence of products, services, and attribute performance on satisfaction. The influence of low performance on overall customer satisfaction was found to be greater than that of high performance, and utility-preserving attributes were applicable to the S-shaped value function curve in prospect theory. Yoshimitsu, Hara, Arait, and Shimomura [57] integrated Kano’s model with prospect theory and proposed a method for assessing customer satisfaction. Tontini et al. [54] considered the psychology of the problem and concluded that low must-be quality performance led to customer dissatisfaction even if attractive and one-dimensional qualities had high performance.

Sampson and Showalter [58] investigated a school lunch program and concluded that the importance and performance of quality attributes was not a point estimation function but a performance-importance response function. Thus, low performance indicated high importance, and the importance level changed when the performance level changed. Customers’ preferences generally change when solutions, contexts, and frames change. People’s preference for risk varies depending upon their reference point and is nonlinear. Risk preferences for loss and gain were found to be asymmetric, with people more sensitive to loss than to gain [56, 59–62]. Therefore, the influence of quality attribute performance on customer satisfaction varies depending on customer reference point, and customer satisfaction is sensitive to performance increase and decrease by different degrees.

Maslow [63] proposed a theory of human motivation, and indicated that human needs are hierarchical with basic needs requiring satisfaction before higher-level needs will appear. Similarly, customers have basic (explicit), expected, and implicit needs for product quality. Needs for must-be qualities are basic needs, needs for one-dimensional qualities are expected needs, and needs for attractive qualities are implicit needs. Tontini et al. [54] indicated that, even if attractive and one-dimensional qualities perform highly, low performance of must-be qualities can lead to customer dissatisfaction. Therefore, must-be quality performance must be high before excellent attractive or one-dimensional qualities will affect customer satisfaction. Customers are more sensitive to quality attributes that dissatisfy them than to those that satisfy them, and thus it is the quality attributes that dissatisfy customers that should be first improved. During product development, the relationship between quantitative evaluation and customer satisfaction should be considered, and the improvement of which attributes should be prioritized must be considered from a psychological perspective.

## Methodology

### Logistic regression analysis

This study employed logistic regression analysis to estimate the odds ratio of customer satisfaction to customer dissatisfaction due to quality element performance. Previous studies have compared the positive influence of high quality attribute performance on customer satisfaction with the negative influence of low quality attribute performance [64–71]. These positive and negative influences explained the asymmetric relationship but did not explain its nonlinearity. The present study deployed logistic regression to describe the nonlinear relationship, to infer the odds ratio of customer satisfaction to customer dissatisfaction due to quality element performance, and to analyze the influence of quality element performance on customer satisfaction. This method explained both the asymmetric relationship and the nonlinear model.

### The proposed approach

According to these considerations, our procedure for evaluating customer satisfaction is as follows:

Step 1: Questionnaire design

Penalty and reward contrast analysis (PRCA) was used for the questionnaire design [18, 22]. A 9-point scale was employed to assess the performance of each quality element (1 = the lowest performance, 9 = the highest performance). The more scale steps, the greater validity of scales [72]. Thus, a scale of 1 to 100 was used to assess customer satisfaction in this study.

Step 2: Data analysis

Logistic regression was used to analyze the odds ratio of high or low performance on each quality element influencing customer satisfaction. Fema and French [73] proposed the three-factor model, a regression model, to assess returns on capital assets. On the basis of this model, we divided customers into two groups according to overall customer satisfaction (i.e., a customer dissatisfaction group and a customer satisfaction group). Quality elements were divided into three groups according to quality element performance, and collected data were coded. A dummy variable was employed for the logistic regression analysis.

Step 3: Classification of quality attributes

Assuming that *k* independent variables exist, the logistic regression equation is

$$p_{i}=\frac{e^{\alpha +\sum_{k=1}^{k}\beta_{k}x_{ki}}}{1+e^{\alpha +\sum_{k=1}^{k}\beta_{k}x_{ki}}}$$ (1)

where *p*_*i*_ = *p*(*y*_*i*_ = 1|*x*_1*i*_,*x*_2*i*_,⋯,*x*_*ki*_) denotes the probability of an event *p*_*i*_; *x*_*ki*_ is the *i*th independent variable; *y*_*i*_ is the *i*th dependent variable; *α* is the intercept and *β*_*k*_ is the *k*th regression coefficient.

The odd ratio *OR*_*i*_ of independent variables is given by

$$OR_{i}=\frac{p_{i}}{1-p_{i}}=e^{\alpha +\sum_{k=1}^{k}\beta_{k}x_{ki}}$$ (2)

**Fig 1:**
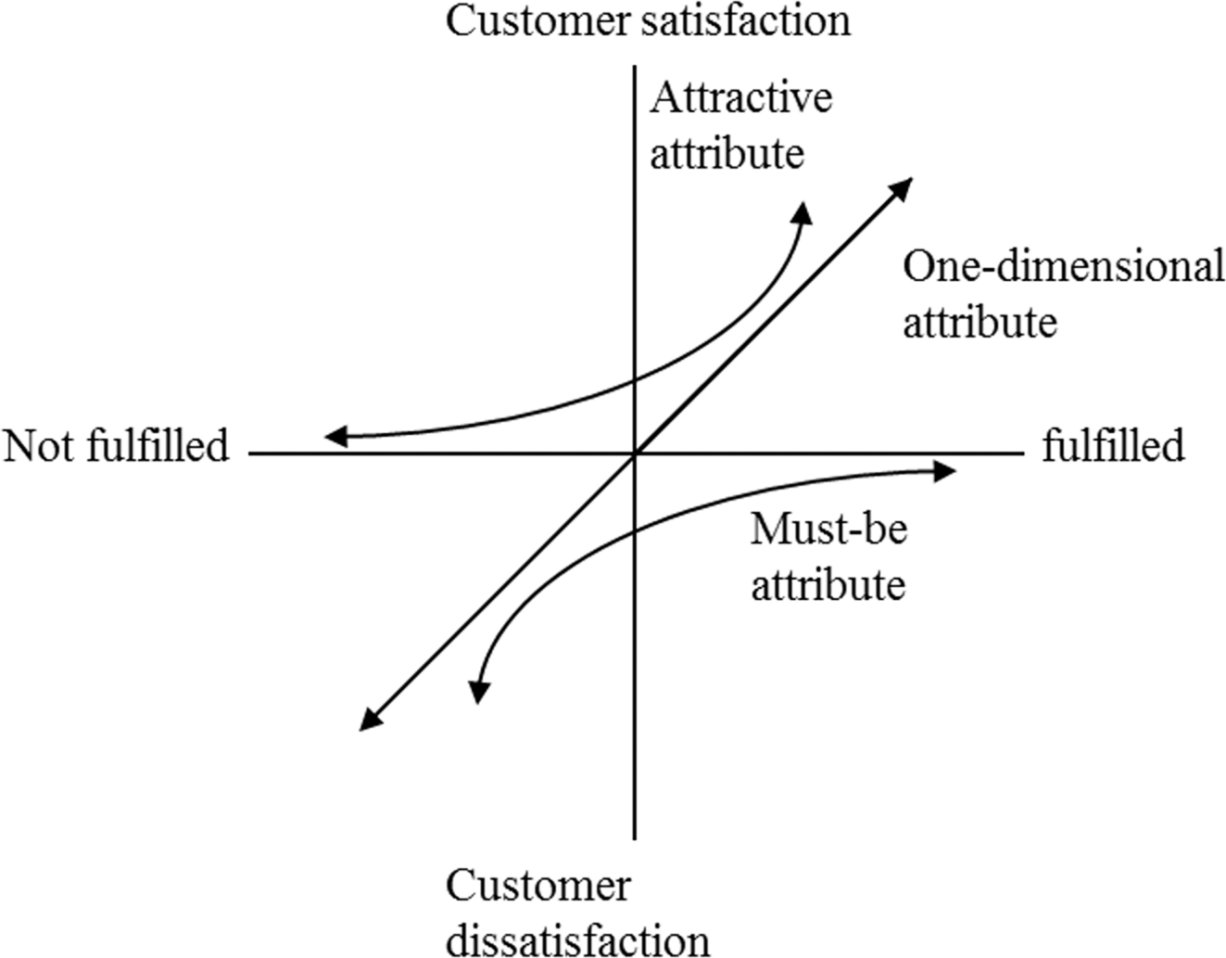
Kano’s model of customer satisfaction.

According to logistic regression analysis, the relationship between quality attribute performance and customer satisfaction is a nonlinear relationship and fits Kano’s model (Fig 1). The customer satisfaction function of an attractive quality attribute is convex, whereas the function of a must-be quality attribute is concave.

The odds ratio was determined using significance and confidence intervals and was employed to classify quality attributes according to the impact of attribute performance on satisfaction, as proscribed in Kano’s model.

Step 4: Decision-making diagram

On the basis of how the customers perceived the functional and dysfunctional quality attributes, the Kano’s model pointed out customer satisfaction was nonlinear function relationship for quality attributes. The Kano category is still qualitative; and further, it hardly estimates the extent of customer satisfaction. Therefore, a decision diagram was employed to prioritize the allocation of resources for improving quality attributes [60,62,74–76].

*ORS*_*i*_ denotes the odds ratio of customer satisfaction, and *ORD*_*i*_ denotes the odds ratio of customer dissatisfaction.

Let *ORS*_*i*_ = tan*θ*′, *π*/4 ≤ *θ*′ < *π*/2
and −*ORD*_*i*_ = −tan*ω*′, *π*/4 ≤ *ω*′ < *π*/2.
Thus, 0 ≤ 2*θ*′−*π*/2 < *π*/2
and 0 ≤ 2*ω*′−*π*/2 < *π*/2.
Let *θ* = 2*θ*′−*π*/2
and *ω* = 2*ω*′−*π*/2.
Thus, *θ*_*i*_ = 2tan^−1^(*x*_*i*_)−*π*/2 where 0 ≤ *θ*_*i*_ < *π*/2,
and *ω*_*i*_ = 2tan^−1^(*x*_*i*_)−*π*/2, where 0 ≤ *ω*_*i*_ < *π*/2,
with *x*_*i*_ = *ORS*_*i*_, *x*_*i*_ ≥ 0; and *x*_*i*_ = *ORD*_*i*_, *x*_*i*_ < 0, respectively.

Therefore,

$$y_{i}=\{\begin{array}{ccc} x_{i}\tan(\pi -2\theta_{i}) & if & x\geq 1\\ -x_{i}\tan(\pi -2\omega_{i}) & if & x<1 \end{array}$$ (3)

**Fig 2:**
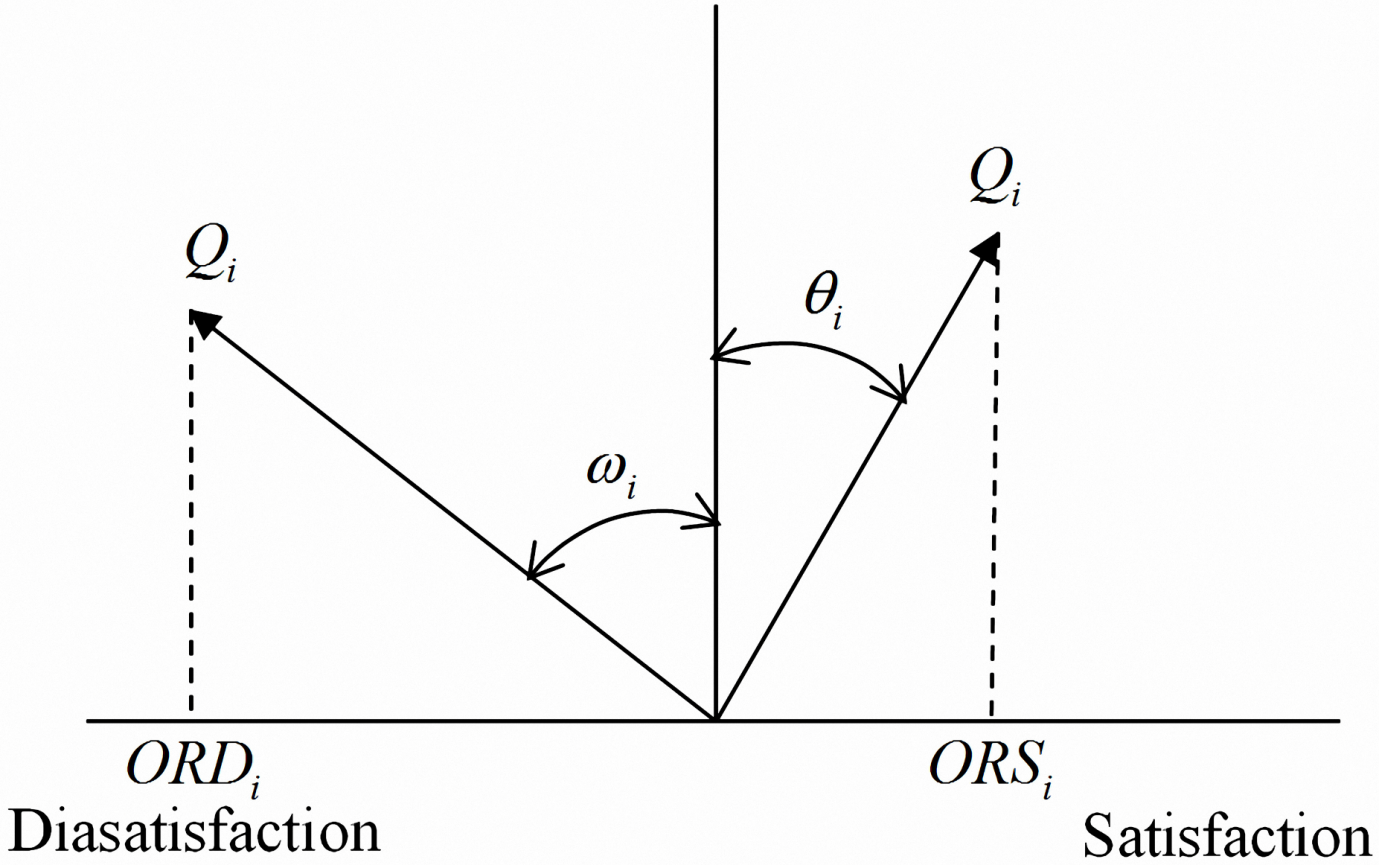
Odds decision diagram for customer satisfaction.

As shown in Fig 2, the angle *θ*_*i*_, formed by the quality attribute vector and the vertical axis, was obtained from the inverse tangent function of *ORS*_*i*_ and denotes the customer satisfaction index. The angle *ω*_*i*_, formed by the quality attribute vector and the vertical axis, was obtained from the inverse cotangent function of *ORD*_*i*_, and denotes the customer dissatisfaction index. The right and left sides of the horizontal axis in Fig 2 represent customer satisfaction and customer dissatisfaction, respectively. The horizontal component of the quality attribute vectors denote the satisfaction odds ratio and the dissatisfaction risk ratio, depending whether the vector points right or left, respectively. The difference in the horizontal components of each vector indicates that the relationship between quality attributes and customer satisfaction is asymmetric.

When the quality attribute vector rotates clockwise from the vertical axis and *θ*_*i*_ approximates 0, a low probability of customer satisfaction with quality attributes is indicated. When the angle is close to *π*/2, the probability of customer satisfaction with quality attributes is high. By contrast, when the vector rotates counterclockwise, the probability of customer dissatisfaction with the quality attributes is high.

Step 5: Decision-making analysis

The left and right sides of the decision-making diagram represent the quality attributes that influence customer dissatisfaction and satisfaction, respectively. Considering the decision sequence, the quality attributes on the right side must be maintained before those on the left side can be improved.

## Case study

### The case

Because of recent technical development, bicycles are used not only for transportation but also for sports and leisure. Understanding cyclists’ experiences about bicycle quality helps manufacturers create products conforming to customer needs. In this study, heavy users of bicycles for sports and leisure were recruited, and quality attributes and customer satisfaction were investigated. A total of 192 cyclists participated in this study, each completing the specific bicycle route of a cycling event. The male cyclists participated in this cycling event are more than female ones. Table 2 shows that 91.7% of respondents were male, and 8.3% were female. The major age groups were from 30 to 49. The very high percentage of respondents participated in the cycling event at least once a month. Most respondents had more than one year experience in the cycling event.

**Table 2:** Demographic characteristics of the respondents.

Category	Response	Frequency (*N* = 192)	Percentage (%)
Gender	Male	16	8.3%
	Female	176	91.7%
Age	Less than 19	8	4.2%
	20–29	26	13.5%
	30–39	67	34.9%
	40–49	53	27.6%
	50–59	29	15.1%
	60 or older	9	4.7%
frequency of the cycling event	1 time 1–3 days	20	10.5%
	1 time 4–6 days	9	4.7%
	1 time 1 week	45	23.4%
	1 time 2 weeks	20	10.4%
	1 time 3 weeks	5	2.5%
	1 time 1 month	31	16.1%
	1 time 2 months	17	8.9%
	1 time 3 months	17	8.9%
	1 time more than 3 months	28	14.6%
Experience of the cycling event	Less than 1 year	11	5.8%
	1 year	45	23.4%
	2 years	30	15.6%
	3 years	30	15.6%
	4 years	19	9.9%
	5 years or more than	57	29.7%

**Fig 3:**
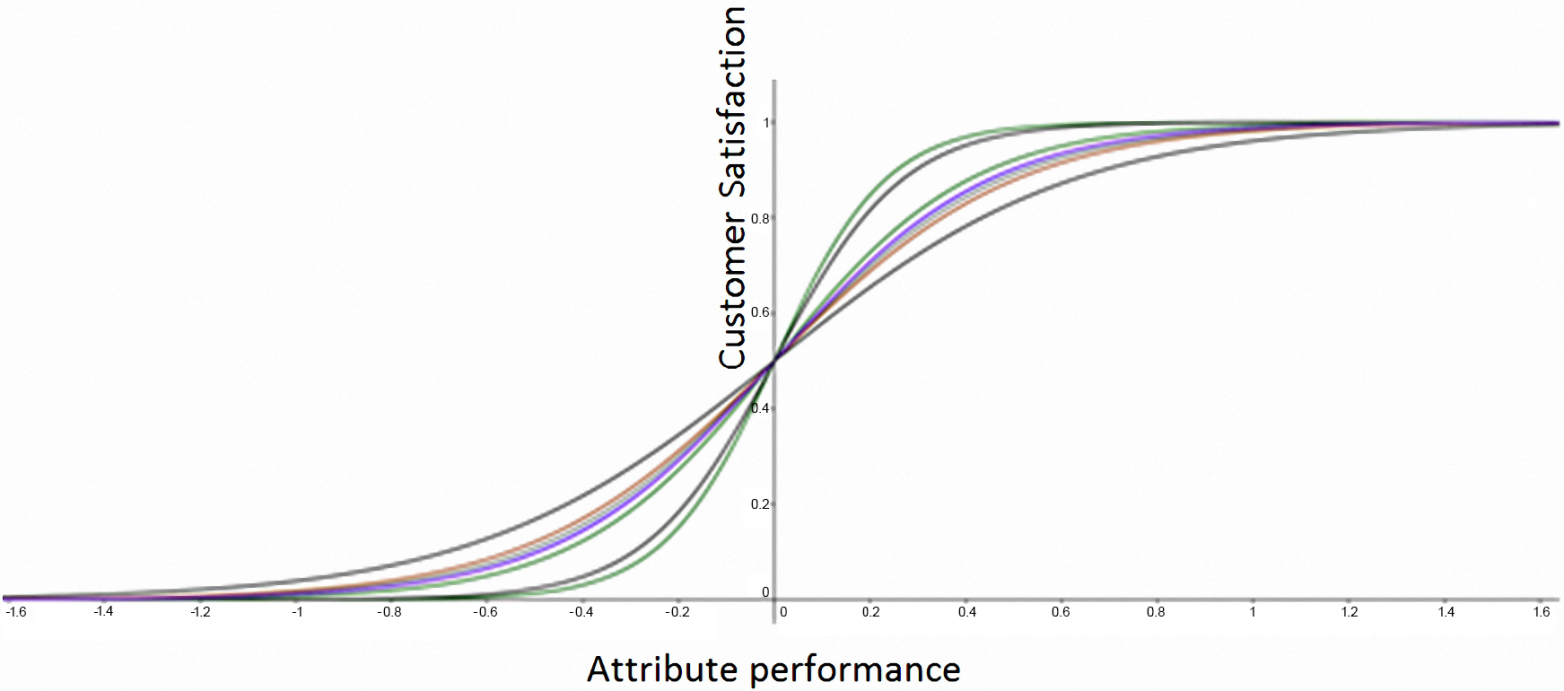
Logistic regressio model for customer satisfaction.

Upon completing the bicycle route, the participants received a questionnaire survey. The PRCA proposed by Brandt [18] was adopted for to design a questionnaire for measuring bicycle-related quality attribute performance and overall customer satisfaction. The questionnaire included eight quality elements: Q1 (appearance), Q2 (color), Q3 (cushion), Q4 (brake system), Q5 (transmission system), Q6 (wheels and transmission system), Q7 (weight), and Q8 (accessories). A 9-point scale was employed to assess quality attribute performance (1 = the lowest, and 9 = the highest performance), and a scale of 1 to 100 was used to assess a cyclist’s satisfaction with their bicycle. The participants’ perceived performance of their bicycle, categorized by the 25^th^ and 75^th^ percentiles into low, medium, and high performances, served as the independent variable, whereas overall satisfaction served as the dependent variable, and the mean value of overall satisfaction was used to divide the participants into two groups: the satisfaction and dissatisfaction groups. If the odds ratio exhibited a significant confidence interval lower limit of >1, then the customers were likely to be satisfied with the high performance of the quality elements or dissatisfied with the low performance of the quality elements. Fig 3 illustrates the logistic function of each quality element. Ranking the quality elements according to the steepness levels of the regression curves revealed a descending order of Q1, Q2, Q5, Q7, Q6, Q3, Q4, and Q8; the steepness level corresponded to the size of the odds.

### Reliability and validity analysis

The properties of measurement scales were tested for internal consistency using Cronbach’s α. The result shows that Cronbach’s α coefficient was 0.949, which is above the benchmark of 0.70 suggested by Nunnally [77]. Therefore, the result suggests a high internal consistency of survey measures. Convergent validity was assessed with confirmation factor analysis (CFA). The standardized factor loadings ranged from 0.747 to 0.890, respectively, which are above the benchmark of 0.60 suggested by Bagozzi and Yi [78]. Hence, convergent validity of the measurement indicators was supported.

#### Statistical analysis and classifications

For each element, two regression coefficients were used to estimate the odds ratios of customer satisfaction and customer dissatisfaction. The odds ratios was then given by Eq (2). Table 3 shows the odds ratios of customer satisfaction and customer dissatisfaction for each quality element. The *R*^2^ value represents the proportion of variance explained by the independent [79]. Model diagnostics were adequate at the Nagelkerke *R*^2^ value of 0.430. For high Q1 performance, the odds ratio of customer satisfaction was 8.600, with a significant confidence interval lower limit >1, indicating that when the appearance performance was high, the likelihood of customer satisfaction was 8.600 times higher than when the performance was low. For low Q1 performance, the odds ratio of customer dissatisfaction was 2.440, indicating that when the appearance performance was low, the likelihood of customer dissatisfaction was 2.440 times higher than when the performance was high. Therefore, if Q1 was adequate or better, customers would be satisfied; otherwise, customers would be dissatisfied; in other words, Q1 was a one-dimensional quality attribute. When Q4, Q5, and Q6 presented high performance, they influenced customer satisfaction to the same degree but with some variation in the likelihood of customer satisfaction. Q4, Q5, and Q6 were also one-dimensional quality attributes. For high Q2 performance, the odds ratio of customer satisfaction was 7.448, indicating that when the color performance was high, the likelihood of customer satisfaction was 7.448 times higher than when the performance was low. For low Q2 performance, the odds ratio of customer dissatisfaction was 1.501, but the lower limit of the confidence interval was 0.722 and was nonsignificant; hence, low Q2 performance did not significantly influence customer dissatisfaction. In other words, if Q2 was adequate, customers would be satisfied; however, inadequate Q2 did not significantly influence customer dissatisfaction, implying Q2 as an attractive quality attribute. Similarly, Q3, Q7, and Q8 were also determined to be attractive quality attributes.

**Table 3:** The odds ratio of attribute-level performance against customer satisfaction.

Quality elements	Mean(S.D.)	High performance (Satisfaction)		Low performance (Dissatisfaction)	
		Odds ratio	95% CI	Odds ratio	95% CI
Q1 appearance	6.781(1.557)	8.600***	[3.320, 22.274]	2.440*	[1.167, 5.103]
Q2 color	6.890(1.441)	7.448***	[3.235, 17.147]	1.501 (ns)	[.722, 3.119]
Q3 cushion	6.635(1.452)	4.195**	[1.614, 10.904]	1.000 (ns)	[.340, 2.939]
Q4 brake system	6.760(1.474)	3.961***	[1.823, 8.604]	2.079*	[1.016, 4.254]
Q5 shift system	6.745(1.511)	4.906***	[2.112, 11.397]	2.675**	[1.303, 5.493]
Q6 wheel set and transmission	6.828(1.446)	3.476**	[1.602, 7.542]	4.414***	[1.997, 9.758]
Q7 weight	6.557(1.485)	4.457**	[1.471, 13.506]	1.220 (ns)	[.365, 4.079]
Q8 accessory	6.604(1.447)	3.196*	[1.254, 8.143]	1.371 (ns)	[.477, 3.947]
overall satisfaction	82.609(12.463)				

Notes: *ns* = not significant. *CI* = confidence interval

*p<0.05.

**p<0.01.

***p<0.001.

Table 4 shows the classification of quality attributes based on the odds ratio of customer satisfaction to customer dissatisfaction. Q1, Q4, Q5, and Q6 influenced not only customer satisfaction, but also customer dissatisfaction.

**Table 4:** Quality attributes categories.

Quality elements	Quality attribute category
Q1 appearance	O
Q2 color	A
Q3 cushion	A
Q4 brake system	O
Q5 shift system	O
Q6 wheel set and transmission	O
Q7 weight	A
Q8 accessory	A

**Fig 4:**
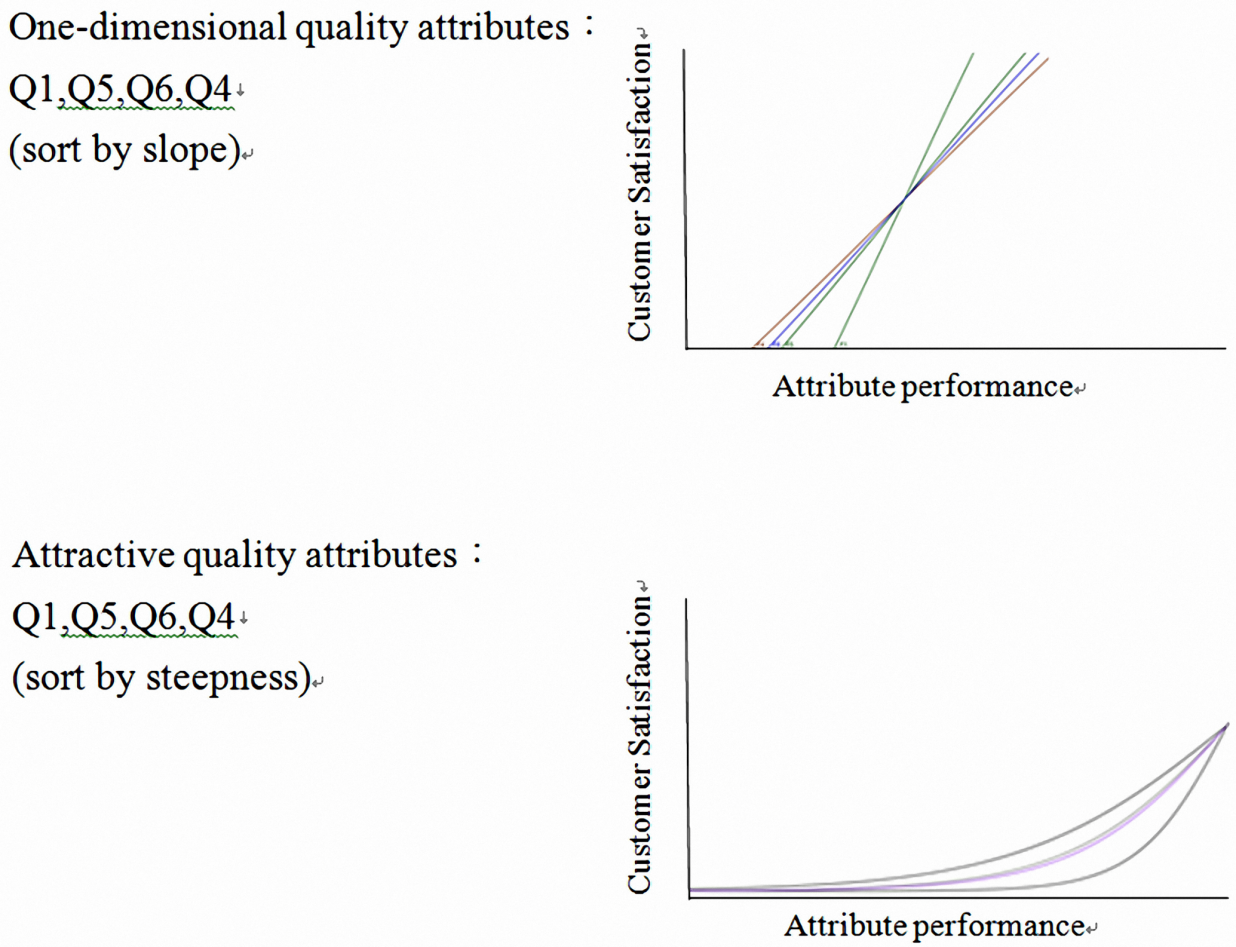
Fitting the quality attributes of Kano’s model.

The quality attribute curves for fitting Kano’s model, as displayed in Fig 4, and their slopes indicated the size of the odds ratio. The one-dimensional quality attributes were Q1, Q5, Q6, and Q4, and the attractive quality attributes were Q2, Q3, Q7, and Q8.

Bicycle users do not appear to have high expectations toward bicycle color, cushion, weight, and accessories, because when these attributes were considered, inadequate quality did not lead to customer dissatisfaction. If these attributes are improved, however, customer satisfaction will still be enhanced. By contrast, the appearance of bicycles has changed over the decades and has been considered crucial by bicycle users. Therefore, adequate appearance leads to customer satisfaction and inadequate appearance leads to customer dissatisfaction. Excellent brake and transmission systems, wheels, other basic bicycle attributes, and excellent bicycle performance are also crucial for customers to enjoy their bicycles and should thus be as high as possible.

### Decision-making analysis

The odds ratios of customer satisfaction (*ORS*_*i*_) and customer dissatisfaction (*ORD*_*i*_) were converted into the customer satisfaction index (*θ*_*i*_) and the customer dissatisfaction index (*ω*_*i*_), respectively, the values of which are listed in Table 5.

**Table 5:** The customer satisfaction index of attribute-level performance.

Quality elements	Customer satisfaction index	
	Satisfaction *θ*_*i*_ (High performance)	Dissatisfaction *ω*_*i*_ (Low performance)
Q1 appearance	1.339	-0.793
Q2 color	1.304	ns
Q3 cushion	1.103	ns
Q4 brake system	1.076	-0.674
Q5 shift system	1.169	-0.855
Q6 wheel set and transmission	1.011	-1.125
Q7 weight	1.129	ns
Q8 accessory	0.964	ns

**Fig 5:**
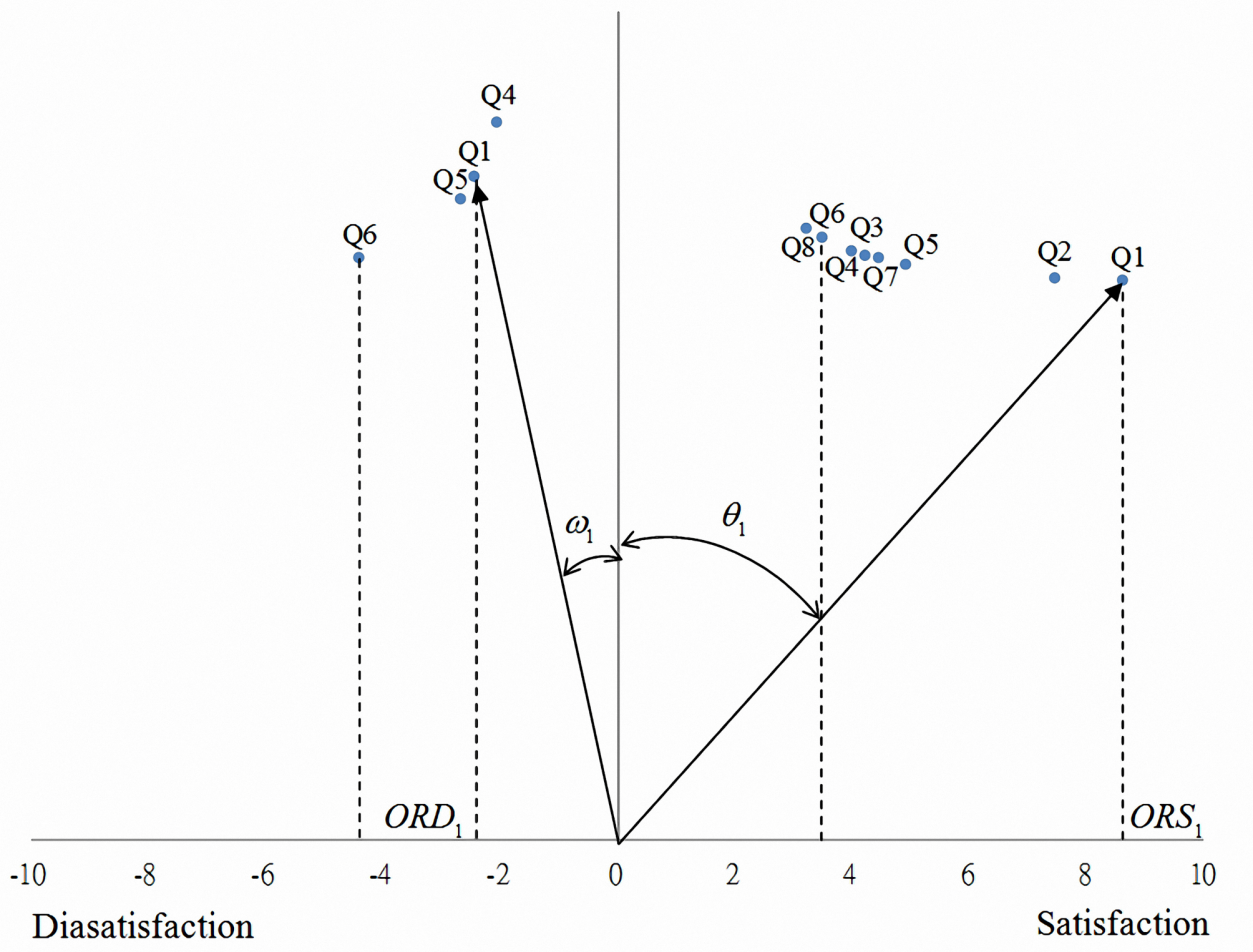
Decision analysis diagram for customer satisfaction odds.

Fig 5 plots the odds of customer satisfaction for quality elements based on the data in Tables 3 and 5. The customer dissatisfaction index was highest for Q6, implying that inadequate quality performance in this attribute led to the maximum customer dissatisfaction. For Q6, the satisfaction and dissatisfaction quality vectors had similar lengths and were approximately symmetrical. Therefore, the Q6 performance should first meet customers’ requirements and then be improved to enhance customer satisfaction. The customer satisfaction index for Q1 was the highest. Thus, customer satisfaction can be enhanced the most by improving Q1 (appearance). The satisfaction and dissatisfaction vectors where asymmetric for Q1 (appearance), demonstrating that the influence of performance on customer satisfaction was greater than that on customer dissatisfaction. The customer satisfaction index for Q2 (color) was the second highest. No dissatisfaction vector was found, indicating an extremely asymmetric relationship wherein the performance of Q2 did not influence customer dissatisfaction. By considering a vector sweeping clockwise around the origin from the horizontal axis, the quality elements that led to customer dissatisfaction are arranged in a descending order. Examining the vertical axis Fig 5 showed that the satisfaction index of the quality elements that led to customer satisfaction increased; therefore, this figure clearly illustrates how the quality elements influenced customer satisfaction, and can thereby facilitate decision analysis.

## Discussion

Logistic regression analysis was used to demonstrate that the relationship between quality attributes classified according to product natures and customer satisfaction was nonlinear. The odds ratio of customer satisfaction was derived, which provides useful information to decision-makers on how to develop their product. The attractive quality attributes, arranged in descending order of customer satisfaction odds ratio, were Q2, Q7, Q3, and Q8. Compared with improvement in Q8 (accessories) performance, improvement in Q2 (color) performance increased the likelihood of customer satisfaction by a factor of 4.252. Therefore, improving color performance is more useful than improving accessories performance. Moreover, with respect to improving Q8, improvement in Q1 attained a greater increase in customer satisfaction odds ratio (by a factor of 5.404) than did improvement in Q2, indicating that improving Q1 performance was more useful than improving Q2 (color) performance (i.e., attractive quality attributes). High performance of attractive quality attributes did not necessarily increase the odds ratio of customer satisfaction, but high one-dimensional quality attribute performance did sometimes enhance customer satisfaction. Compared with improving quality attributes from the perspective of quality attribute classification, considering the odds ratio of customer satisfaction to improve quality attributes was more likely to win customers. To reduce costs and avoid customer dissatisfaction, the attribute Q6 (wheels and transmission system), which attained the highest dissatisfaction odds ratio, should be considered first. By examining the odds ratios of customer satisfaction and dissatisfaction, decision-makers can perform a quantitative classification to identify what factors enhance customer satisfaction. When customers’ requirements are not met, quality attributes with high dissatisfaction odds ratios should be improved, whereas to further enhance customer satisfaction, quality attributes with high satisfaction odds ratios should be enhanced.

**Fig 6:**
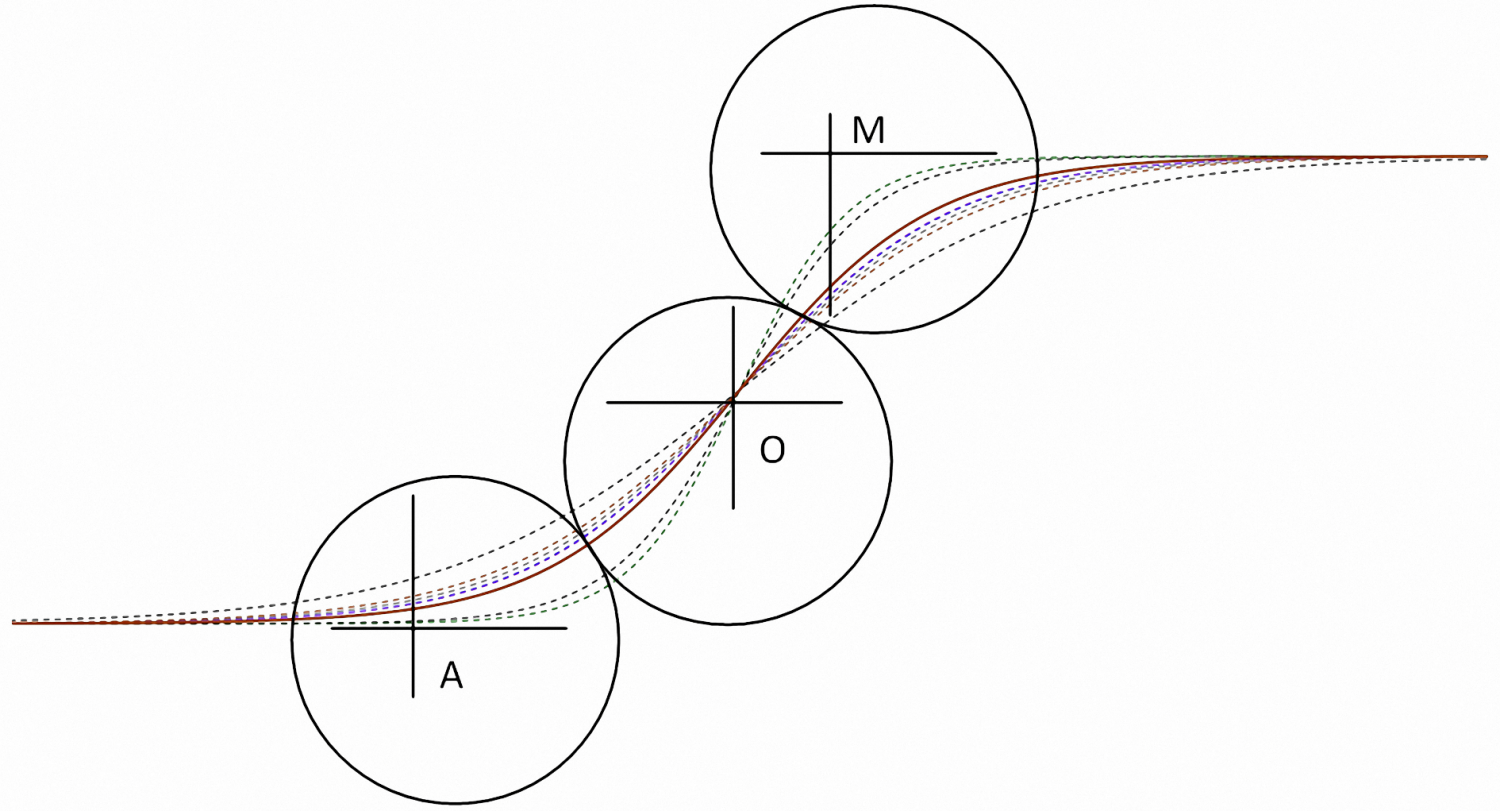
Quality attributes and customer satisfaction.

Illustrated in Fig 6, the life cycle curve of overall quality attributes was derived from the geometric mean of the quality element odds ratios; the dotted lines represent each quality element, and the solid line represents the overall quality attribute. According to Kano [80], over the life cycle of an attribute, it will begin as an attractive quality, then progress to being a one-dimensional quality, and then finally it will become a must-be quality. As quality attributes change, the reference point of customer satisfaction with quality attribute performance changes. High and low performances influence customer satisfaction differently. A life cycle curve can be used to assess the advantages and disadvantages of enhancing and reducing attribute performances.

**Fig 7:**
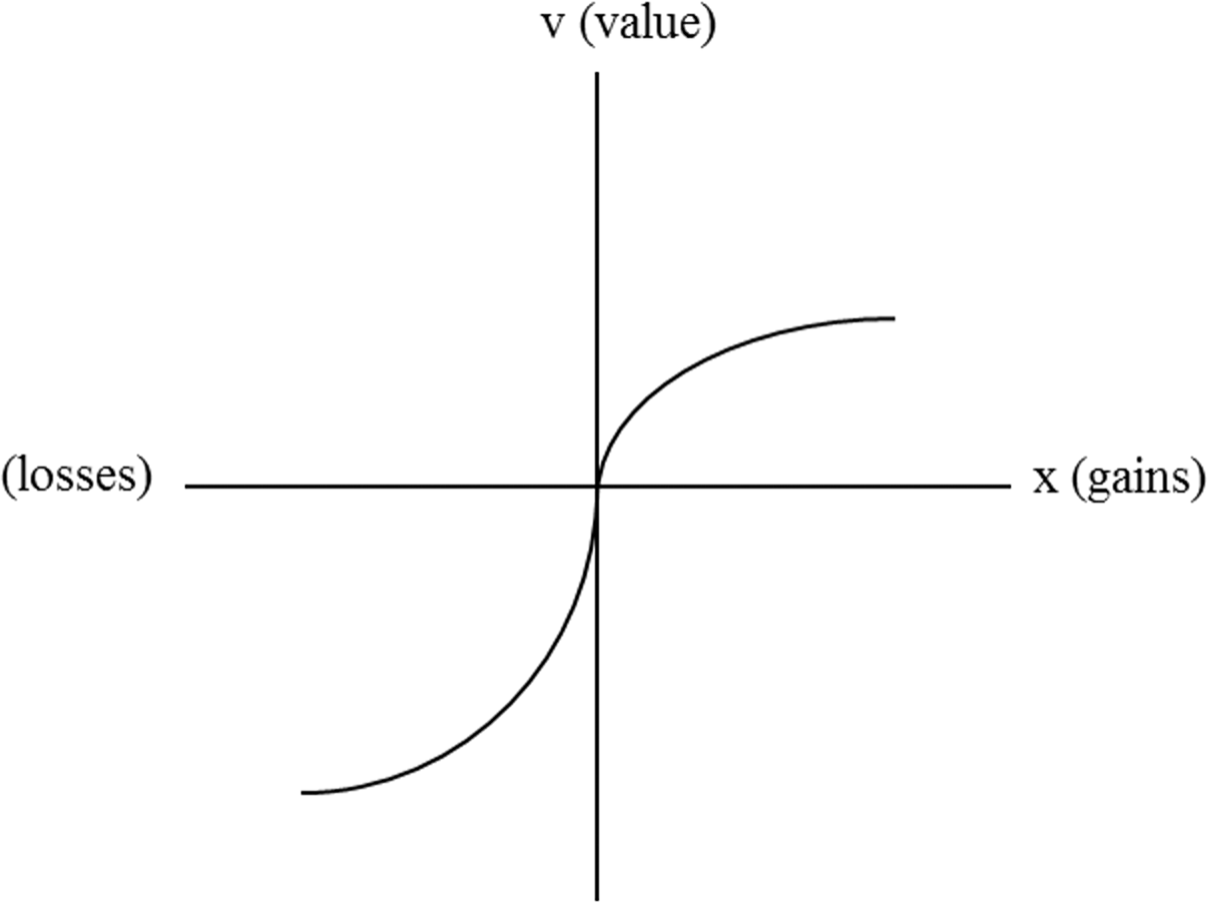
Value function of prospect theory.

Neumann and Morgenstem [81] proposed expected utility theory to describe rational decision behavior and explore the relationship between wealth level and utility. Allais [82] proposed the Allais paradox, which contradicted expected utility theory. Kahneman and Tversky [55] explored decision-making behavior according to psychology and proposed prospect theory, which posited that customers typically adopt a relative method of thinking and consider that value is determined by wealth change instead of wealth level (Fig 7). For gain, a concave function is obtained; for loss, a convex function is obtained. The expected utility theory does not explain why people prefer certainty, whereas prospect theory does.

The value function is defined as follows:

$$v\left(x\right)=\left\{ \begin{array}{ccc} x^{\alpha } & if & x\geq 0\\ -\lambda {\left(-x\right)}^{\beta } & if & x<0 \end{array}\right.$$ (4)

where *α* and *β* are between 0 and 1; *λ* > 1; *x* > 0 indicates gain; and *x* < 0 indicates loss. For our results, *α = β* ≈ 0.88 and *λ* ≈ 2.25 [83]. According to Kano’s model, quality element performance influences customer satisfaction. Similarly, according to expected utility theory, wealth level influences wealth utility. Moreover, according to prospect theory, value is determined using wealth change, not wealth level. When decision-making behavior is explained from a psychology perspective, prospect theory can be used to explain how quality attribute performance determines customer satisfaction.

The odds ratio for quality elements is $OR=e^{\alpha^{\prime }+\beta^{\prime }x^{\prime }}$.

When quality element performance changes by one unit, *x*′ = 1 and the odds ratio can be expressed as follows: $OR=e^{\alpha^{\prime }+\beta^{\prime }}$.

The odd ratio for quality element *i* is $y_{i}^{\prime }=e^{\alpha^{\prime }+\beta_{i}^{\prime }}$.

The change in customer satisfaction due to a change in quality element performance is expressed as

$$\frac{dy^{\prime }}{d\beta^{\prime }}=e^{\alpha^{\prime }+\beta_{i}^{\prime }}.$$

Therefore, the change in quality element performance can cause a gain in customer satisfaction and a loss of customer dissatisfaction.

Let $x=e^{\alpha^{\prime }+\beta_{i}^{\prime }}$ where *x* denotes gain and loss in prospect theory. The value function *v*(*x*) is the value produced by the gain and loss. Table 6 details the value derived from the change in quality attribute performance. When the gain increased by one unit, the increase in value decreased, and vice versa. However, compared with gain for attractive quality attributes, must-be quality attributes were more sensitive to loss.

**Table 6:** Customer satisfaction value.

Quality elements	Value	
	Gain	Loss
Q1 appearance	6.643	-4.933
Q2 color	5.853	
Q3 cushion	3.532	
Q4 brake system	3.358	-4.284
Q5 shift system	4.054	-5.348
Q6 wheel set and transmission	2.993	-8.311
Q7 weight	3.725	
Q8 accessory	2.780	

Analyzing how improvement in quality attributes changes customers’ perceived value enables identifying the quality attributes that offer the maximum value. This will help decision-makers determine how customer satisfaction influences value creation, how to improve quality attributes, and which quality attributes are the highest priority.

## Conclusions

In the present study, a novel method was proposed to quantitatively examine the asymmetrical and nonlinear relationship between quality attributes and customer satisfaction, and to classify quality attributes on the basis of this relationship. To sum up, these findings are not only that the influences of some attributes on customer satisfaction are significant, but that the logistic regression models the probability of customer satisfaction to fit the nonlinear relationship. Examining the cyclists confirmed that quality attributes and customer satisfaction were in an asymmetric and nonlinear relationship, as found in previous studies [51, 20, 24]. Berger, et al. [84] proposed the customer satisfaction index to indicate the extent of customer satisfaction. The customer satisfaction coefficient is measured by the frequency of the quality attribute. However, this study employed the odds ratio to estimate the extent of customer satisfaction. It appears the logistic regression method produces more accurate measurements and more useful information. Utilizing the shape changes of the nonlinear relationship encourage strategic thinking to optimize customer satisfaction through improving the performance of various attribute. In addition to the likelihood of customer satisfaction, this method is more strongly linked to success in the nonlinear relationship than the previous methods. The value function calculated using prospect theory revealed an S-shaped curve, and could be used to analyze customer satisfaction. Resources and production capacity are generally limited during product design and manufacture, so by considering customer psychology and the asymmetric and nonlinear relationship between attributes and satisfaction, time and money need not be wasted on product changes that will not increase customer satisfaction.

Kano’s model is constructed according to customers’ requirements, which change with time and situation [11, 85]. The reference point used by customers to assess quality attribute performance often changes. A decision-making diagram was presented in this study, visualizing how quality attributes affect customer satisfaction. The decision-making diagram correspond to the life cycle of quality attributes proposed by Kano [8]. Previous studies reported that for successful quality attributes, an indifferent quality changes to an attractive quality over time, which in turn becomes a one-dimensional quality and finally a must-be quality [86–88]. Thaler [89] proposed the endowment effect, which states that when a person owns an object, they want to avoid losing it. Therefore, when a customer owns a quality element, the customer wants to avoid losing the quality element. Slack [90] reported that if the importance of a quality attribute increased (i.e., customers came to value it more highly), then customers would cease to be easily satisfied because the quality is in the process of transforming from an attractive quality into a must-be quality. For example, Kano [80] investigated TV remote controls, which were considered as an attractive quality in 1983, a one-dimensional quality in 1989, and a must-be quality in 1998. The reference point used by customers to assess the quality of a TV remote control changed with time. Customers typically avoided losing existing quality attributes, and changed their decision-making preference from gain to loss, demonstrating that quality attributes are not fixed, but dynamic.

For bicycles, the quality attribute life cycle is long, but for mobile phones, for example, the quality attribute life cycle is short. The development of quality attributes must be understood if products are to be manufactured that satisfy customers. In the present study, we not only classified quality attributes and examined the change of quality attributes, but also quantitatively analyzed a dynamic two-dimensional quality model. Subsequent studies are suggested to examine quality attribute life cycles through exploring the impact of performance change on customer satisfaction, which predicts changes in quality attributes. In the present study, the proposed novel method employs the concept of customer satisfaction odds. Comprehensive and profound empirical studies remain to be conducted to demonstrate the applicability of this method.

## Supporting information

S1 File Questionnaire–English. (DOCX)

S2 File Questionnaire–Chinese. (DOCX)
