# Supplementary material for: Empirical research on Kano’s model and customer satisfaction
Source: PLoS One. 2017 Sep 5;12(9):e0183888. doi: 10.1371/journal.pone.0183888 (PMC5584930; doi:10.1371/journal.pone.0183888)
Supplement: S1 File — (DOCX) [file pone.0183888.s001.docx]

**Exploring the Bicycle-related Quality Attribute Evaluation**

**Questionnaire Survey**

Dear Participants:

We are inviting you to participate in a research study. In our study, we are exploring the bicycle-related quality attribute evaluation. Please complete this assessment questionnaire. Your responses to this survey will be used only for academic research purposes and kept confidential and anonymous. By submitting this survey, you are indicating your consent to participate in the study. Your participation is appreciated.

Ph.D. Program of Technology Management, Chung Hua University, Hsinchu, Taiwan

Feng-Han Lin

Part І Demographic Characteristics

1.What is your gender?

□ Male □ Female

2.What is your age?

□ Less than 19 □ 20~29 □ 30~39 □ 40~49 □ 50~59 □ 60 or more than

3. How often do you participate in the cycling event?

□ 1 day □ 2~3 days □ 4~6 days □ 1week □ 2 weeks □ 3 weeks
□ 1 month □ 2 months □ 3 months □ more than 3 months

4. How long have you participated in the cycling event?

□ Less than 1 year □ 1 year □ 2years □ 3 years □ 4 years
□ 5 years or more than

Part II Questionnaire content

How do you feel the following attributes of the bicycle and overall satisfaction? (Please tick boxes below and score overall satisfaction.)

| Attributes | **Extremely dissatisfied** | | | **dissatisfied** | | **Neutral** | | | **Satisfied** | | **Extremely satisfied** | |
| --- | --- | --- | --- | --- | --- | --- | --- | --- | --- | --- | --- | --- |
|  | 1 | 2 | 3 | | 4 | | 5 | 6 | | 7 | 8 | 9 |
| 1.appearance | □ | □ | □ | | □ | | □ | □ | | □ | □ | □ |
| 2.color | □ | □ | □ | | □ | | □ | □ | | □ | □ | □ |
| 3. cushion comfort | □ | □ | □ | | □ | | □ | □ | | □ | □ | □ |
| 4. brake system | □ | □ | □ | | □ | | □ | □ | | □ | □ | □ |
| 5. shift system | □ | □ | □ | | □ | | □ | □ | | □ | □ | □ |
| 6.wheel set  and transmission | □ | □ | □ | | □ | | □ | □ | | □ | □ | □ |
| 7.weight | □ | □ | □ | | □ | | □ | □ | | □ | □ | □ |
| 8.accessories | □ | □ | □ | | □ | | □ | □ | | □ | □ | □ |

Please evaluate overall satisfaction on a scale of 1–100 for your bicycle.

What's the score?
