# Supplementary material for: Empirical research on Kano’s model and customer satisfaction
Source: PLoS One. 2017 Sep 5;12(9):e0183888. doi: 10.1371/journal.pone.0183888 (PMC5584930; doi:10.1371/journal.pone.0183888)
Supplement: S2 File — (DOCX) [file pone.0183888.s002.docx]

**自行車之研究調查**

| 您好，邀請您參與我們對於自行車的品質屬性評估之研究調查。懇請依實情程度來回答評估問卷，以提高本問卷之實務參考價值。此份問卷以不具名，僅作為實務分析不涉及任何商業利益。在您完全自主同意參加這次研究調查之下，請提交問卷。感謝您撥冗參與這次問卷研究。  中華大學科技管理博士學位學程  林豐涵 |
| --- |

**一、基本資料**

以下為基本資料，請您依序勾選

1.性別：□男□女

2.年齡：□19歲以下□20~29歲□30~39歲□40~49歲□50~59歲□60歲以上

3.請問最近一年您多久參加一次自行車社團活動? □每天□2~3天□4~6天□1週□2週□3週
□1個月□2個月□3個月□3個月以上

4.在騎乘自行車遊憩已有的資歷：□1年以下□1年□2年□3年□4年□5年以上

**二、問卷**

請您針對此次自行車遊憩活動所騎乘自行車相關要素的品質評價，在您覺得合適的選項中勾選，分數為1~9，並請為自行車的總體滿意度評分，分數為1~100。

| 屬性 | **極不滿意** |  | **不滿意** |  | **普通** |  | **滿意** |  | **極為滿意** |
| --- | --- | --- | --- | --- | --- | --- | --- | --- | --- |
|  | 1 | 2 | 3 | 4 | 5 | 6 | 7 | 8 | 9 |
| 1.自行車的外型 | □ | □ | □ | □ | □ | □ | □ | □ | □ |
| 2.自行車的配色 | □ | □ | □ | □ | □ | □ | □ | □ | □ |
| 3.自行車坐墊的舒適度 | □ | □ | □ | □ | □ | □ | □ | □ | □ |
| 4.自行車剎車系統功能 | □ | □ | □ | □ | □ | □ | □ | □ | □ |
| 5.自行車變速系統功能 | □ | □ | □ | □ | □ | □ | □ | □ | □ |
| 6.自行車輪組與傳動系統 | □ | □ | □ | □ | □ | □ | □ | □ | □ |
| 7.自行車整車的重量 | □ | □ | □ | □ | □ | □ | □ | □ | □ |
| 8.自行車的附件(配件) | □ | □ | □ | □ | □ | □ | □ | □ | □ |

對於**此次自行車遊憩活動所騎乘自行車**總體表現的滿意度分數，以0~100分來評分，您會給 分
